# ‘The phone is my boss and my helper’ – A gender analysis of an mHealth intervention with Health Extension Workers in Southern Ethiopia

**DOI:** 10.1093/pubmed/fdy199

**Published:** 2018-12-14

**Authors:** Rosalind Steege, Linda Waldman, Daniel G Datiko, Aschenaki Z Kea, Miriam Taegtmeyer, Sally Theobald

**Affiliations:** 1 Department of International Public Health, Liverpool School of Tropical Medicine, Pembroke Place, L3 5QA, UK; 2 Institute of Development studies, Library Road, Brighton BN1 9RE, UK; 3 REACH Ethiopia, Hawassa, Ethiopia

## Abstract

**Background:**

There is considerable optimism in mHealth’s potential to overcome health system deficiencies, yet gender inequalities can weaken attempts to scale-up mHealth initiatives. We report on the gendered experiences of an mHealth intervention, in Southern Ethiopia, realised by the all-female cadre of Health Extension Workers (HEWs).

**Methodology:**

Following the introduction of the mHealth intervention, in-depth interviews (*n* = 19) and focus group discussions (*n* = 8) with HEWs, supervisors and community leaders were undertaken to understand whether technology acted as an empowering tool for HEWs. Data was analysed iteratively using thematic analysis informed by a socio-ecological model, then assessed against the World Health Organisation’s gender responsive assessment scale.

**Results:**

HEWs reported experiencing: improved status after the intervention; respect from community members and were smartphone gatekeepers in their households. HEWs working alone at health posts felt smartphones provided additional support. Conversely, smartphones introduced new power dynamics between HEWs, impacting the distribution of labour. There were also negative cost implications for the HEWs, which warrant further exploration.

**Conclusion:**

MHealth has the potential to improve community health service delivery and the experiences of HEWs who deliver it. The introduction of this technology requires exploration to ensure that new gender and power relations transform, rather than disadvantage, women.

**Keywords:**

communities, e-health, gender

## Resumen

**Antecedentes:**

Existe un optimismo considerable con respecto al potencial de mSalud para superar las deficiencias en el sistema de salud, sin embargo, las desigualdades de género pueden debilitar los intentos de ampliar las iniciativas de mSalud. Analizamos las experiencias de género de una intervención de mSalud en el sur de Etiopía que realizó un equipo femenino de agentes de extensión sanitaria (AES).

**Metodología:**

A continuación de la presentación de la intervención de mSalud, se organizaron entrevistas exhaustivas (n = 19) y debates en grupos focales (n = 8) con los AES, los supervisores y los líderes comunitarios para comprender si la tecnología actuaba como una herramienta facultativa para los AES. Los datos se analizaron reiteradamente por medio de análisis temáticos informados por un modelo socio-ecológico, luego se compararon con la escala de evaluación de respuestas basadas en género de la Organización Mundial de la Salud.

**Resultados:**

Los AES experimentaron lo siguiente: un estado mejorado después de la intervención, respecto de los miembros de la comunidad y fueron los encargados de los teléfonos inteligentes en sus hogares. Los AES que se desempeñan solos en puestos de salud sintieron que los teléfonos inteligentes les brindaron ayuda adicional. En cambio, los teléfonos inteligentes presentaron una nueva dinámica de poder entre los AES, impactando la distribución de la labor. También hubo consecuencias negativas relacionadas con el costo para los AES. Esto se debe investigar en profundidad.

**Conclusión:**

MSalud tiene el potencial de mejorar la prestación de los servicios para la salud comunitaria y las experiencias de los AES que los brindan. La presentación de esta tecnología requiere de investigación para garantizar de que las nuevas relaciones de género y poder sean transformadoras para las mujeres, en lugar de perjudicarlas.

**Palabras clave:**

mSalud, ética y comunidades.

## Résumé

**Contexte:**

L’optimisme entourant le potentiel de la santé mobile à remédier aux failles du système de santé est considérable, mais les inégalités entre les sexes peuvent affaiblir les tentatives de mise à l’échelle des initiatives de santé mobile. Nous faisons rapport sur les expériences liées au genre d’une intervention de santé mobiledans le sud de l’Éthiopie, réalisée par un effectif féminin d’agentes de vulgarisation sanitaire (AVS).

**Méthodologie:**

Après l’introduction de l’intervention de santé mobile, des entrevues en profondeur (n = 19) et des discussions de groupe (n = 8) avec des AVS, des superviseurs et des dirigeants communautaires ont été menées pour comprendre si la technologie a servi d’outil d’autonomisation pour les AVS. Les données ont été analysées à l’aide de l’analyse thématique de façon itérative en se basant sur un modèle socio-écologique, puis évaluées en fonction de l’échelle de prise en compte du genre de l’Organisation mondiale de la Santé.

**Résultats:**

Les AVS ont indiqué que leur situation s’est améliorée après l’intervention, qu’elles ont gagné le respect des membres de la communauté et qu’elles étaient des gardiennes des téléphones intelligents dans leur ménage. Les AVS travaillant seules aux dispensaires de soins de santé ont estimé que les téléphones intelligents apportaient un soutien supplémentaire. Par ailleurs, les téléphones intelligents ont introduit de nouvelles dynamiques de pouvoir entre les AVS, ce qui a une incidence sur la répartition du travail. Il y avait aussi des répercussions négatives au chapitre des coûts pour les AVS, ce qui justifie un examen plus approfondi.

**Conclusion:**

La santé mobile a le potentiel d’améliorer la prestation des services de santé communautaire et l’expérience des AVS qui les prodiguent. L’introduction de cette technologie nécessite d’être examinée pour s’assurer que les nouvelles relations de genre et de pouvoir transforment les femmes, plutôt que de leur nuire.

**Mots clés:**

santé mobile, éthique et communautés.

## Introduction

Mobile health (mHealth) provides health services and information via mobile technologies, including mobile phones.^1^ There is considerable optimism in mHealth’s potential to overcome health systems’ deficiencies to ensure access to safe, effective and affordable health services.^2^ This has led to an ‘explosion of mHealth activities’^3^ and ‘large-scale adoption and deployment of mobile phones’^4^ by Community Health Worker (CHW) programmes. MHealth innovation in relation to CHWs, on which low- and middle-income countries (LMICs) disproportionately depend, has been reported to be ‘particularly promising’.^5^ CHWs’ use of mHealth has the potential to improve their motivation; decision-making; training; adherence to guidelines; data entry and quality; planning and efficiency; and communication and health promotion; while also enhancing coverage and timeliness of services and reducing costs.^1,2,5–8^ MHealth also allows the monitoring and tracking of health indicators in real time, providing crucial insights to policy makers and enabling CHWs to better serve communities.^9^

Research on CHWs’ use of mHealth focuses on the formal, work-related aspects, such as health outcomes and/or health system benefits. Recent systematic reviews^1,4,10,11^ note CHWs acceptance of mHealth’s potential to enhance health outcomes and health systems and benefit CHWs. Adoption however, is hindered by infrastructural limitations (e.g. electricity and internet), security issues and a lack of sustainability given that most interventions are pilots. With a few exceptions,^5,12^ CHWs’ perceptions of mHealth, and their positionality as gendered subjects and workers is overlooked. As Lupton (2014) has argued, examination of how CHWs have used mHealth has ‘received almost no attention from critical scholars’.^13^

Sustainable Development Goal 5 calls for ‘*the use of enabling technology, in particular ICTs [Information Communication Technologies], to promote the empowerment of women*’.^14^ Aligned with this, pilot mHealth interventions provide women with greater access to health care information and more autonomy in health decision-making. MHealth technologies for CHWs, an often-feminized and sometimes volunteer cadre, offer a unique opportunity to explore the potential for empowerment. CHWs are crucial lynchpins in LMICs’ health systems, yet experience limited training, heavy workloads and undertake a range of service delivery tasks.^5,15^ Situated within broader socio-cultural and gendered contexts, they are burdened both by their workloads and by gendered roles and responsibilities to kin and communities.^16,17^

**Fig. 1:**
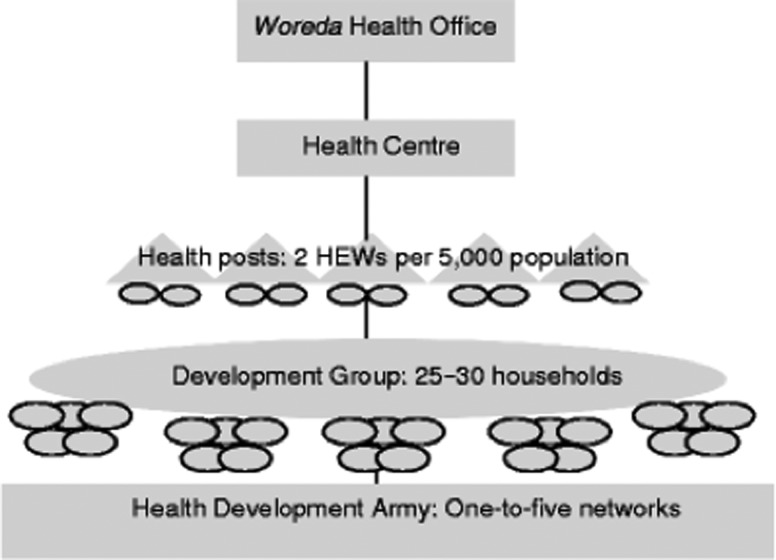
Overview of HEWs’ intermediary position between the community and health sector.

Gender inequalities can weaken attempts to scale-up mHealth and mHealth initiatives do not always lead to women’s empowerment;^2,4,12^ gender transformative initiatives that promote equality and transform gender norms are needed.^12^ Several tools encourage a more gender transformative approach assessing gender norms and power relationships. This includes the World Health Organisation’s (WHO) gender responsive assessment framework,^18^ which helps position a project from gender blind to gender transformative by setting out basic criteria to be met in each category (see Fig. 1). This framework identifies necessary milestones/actions for interventions seeking to achieve gender transformation.

This study explores the impact of an mHealth pilot intervention, a smartphone-based digital health management information system (HMIS),^19^ on Ethiopia’s female HEWs. Framed by a socio-ecological model, and using the WHO’s gender framework, it assesses whether technology acts as an empowering tool for HEWs and how the intended and unintended consequences influence gender and power dynamics.

## Methods

**Fig. 2:**
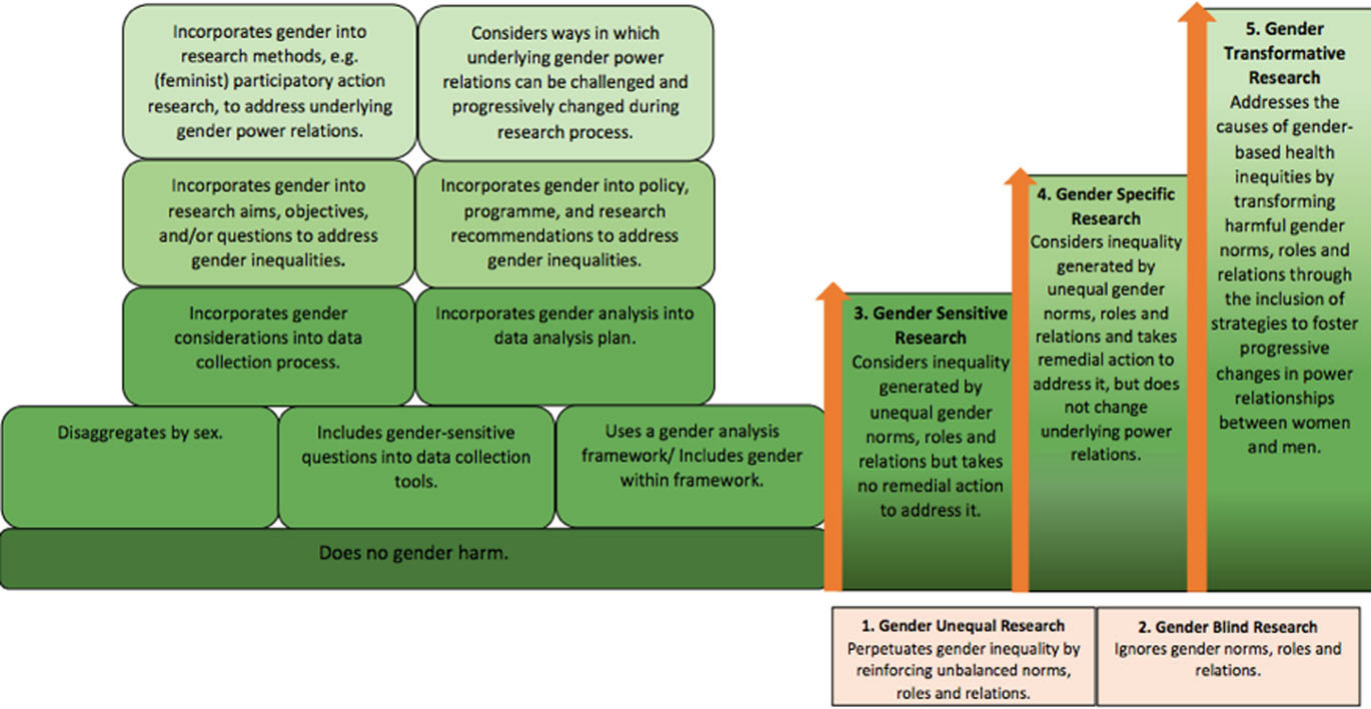
Adapted from the WHO Gender Responsive Assessment Scale: WHO, (2011). Gender mainstreaming for health managers: a practical approach. Geneva.

In Ethiopia, the Health Extension Programme (HEP), initiated in 2004, is a free primary health care package in which 38 000 female HEWs offer 16 essential health packages.^20–22^ HEWs are salaried government employees who have completed at least grade ten. They are selected by their communities to complete one year of training in basic health service delivery. A health post serves a population of about 5000 and is staffed by two HEWs accountable to the *kebele* (lowest administrative unit). HEWs are supported by female volunteers, known as the ‘Health Development Army’^18^ and supervised by health professionals from health centres. Health centres in turn, are overseen by the *woreda* (district) health office (Fig. 2).

In spite of low ICT access and usage compared with other African countries, the Ethiopian Federal Ministry of Health has embraced mHealth in its national strategic health plan.^23^ Ethiopia prioritizes maternal health services and calls for improved HEW performance on maternal health-related tasks.^20,21,24^

### The intervention

An mHealth intervention that focussed on the priority areas of TB and maternal health services^19^ and linked to the Ethiopian Ministry of Health’s mHealth strategic framework was conducted in Sidama zone, Southern Ethiopia, with a population of about 3.7 million. Our research, undertaken in six Primary Health Care Units across six districts, worked closely with and was realized by HEWs, their supervisors, health workers based at the catchment health centres and policy makers at *woreda* health office and zonal health department.

One smartphone, assigned to each health post, was shared between two HEWs, who used the phone to input data on expectant mothers and TB. The data was uploaded to the HMIS where it was instantly available to other levels of the health system. Reminder messages prompted HEWs to follow-up on expectant mothers’ due dates and sputum examination for TB symptomatic cases.

Ninety-seven smartphones and eight computers were distributed to HEWs, their supervisors, health centre staff and focal persons from district and zonal levels. Ongoing theoretical and practical training was conducted and a monthly airtime allowance of 100 birr (3.64 USD) was provided for the first five months. Subsequent top-ups were paid for by HEWs.

### Ethics statement

Ethics was approved by the Liverpool School of Tropical Medicine^16–22^ and by the Ethiopian Ministry for Science and Technology in June 2016, and supported by the Regional Health Bureau. All participants gave written informed consent.

### Data collection process

Qualitative methods were used to generate rich insights into participants’ experiences of the intervention.^25^ They included face-to-face semi-structured in-depth interviews (IDIs, *n* = 19) and single sex focus group discussions (FGDs, *n* = 8) with HEWs, supervisors and community leaders (Table 1). (In the study districts, all HEWs are female and all community leaders male. Supervisors are predominantly male. Disaggregating by gender and district would breech confidentiality.) Interview topic guides explored the gendered elements of the intervention; ways in which the mobile phones helped or hindered HEWs’ roles, how HEWs used the phones outside of work and the impact on their relationships. Analysis, informed by an adapted socio-ecological model, was designed to evaluate how the intervention impacted the interface position of the HEWs and to establish how the intervention fared along the WHO’s gender transformative scale. Interviews were conducted in four districts purposively selected for variation in geographic location and performance.

**Table 1:** Qualitative interviews conducted by participant and district

District participant	District 1	District 2	District 3	District 4
HEW	2 × IDIs (Female)	3 × IDIs (Female)	4 × IDIs (Female)	5 × IDIs (Female)
	1 × FGD (Female)	1 × FGD (Female)^a^		1 × FGD (Female)
HEW Supervisor	1× IDI (Female)	1 × IDI (Male)	1 × IDI (Male)	1 × IDI (Male)
	1 × IDI (Male)	1 × FGD (Male)^b^	1 × FGD (Male)	1 × FGD (Male)
Community leaders	1 × FGD (Male)	1 × FGD (Male)	1 × FGD (Male)	1 × FGD (Male)

^a^Merged with participants from District 3 due to geographical proximity and convenience of participants.

^b^Merged with participants from District 4 due to geographical proximity and convenience of participants.

In interviews, a local trained female research assistant, fluent in Sidamigna (the local dialect), ensured HEWs felt comfortable, and used topic guides to facilitate conversation. The lead researcher (RS) was on hand to clarify any questions or concerns. Interviews were conducted at health posts, health centres and *woreda* health offices, scheduled in private spaces, and recorded. These were transcribed and translated into English. Translation quality was reviewed (AZK). Qualitative analysis was done by reading and re-reading transcripts to identify iterative themes^26^ and select appropriate quotes (RS with inputs from AZK and DGD). Software NVivo was used to code and run queries on the data. Attention was paid to give voice to the majority and minority views.

## Results

**Fig. 3:**
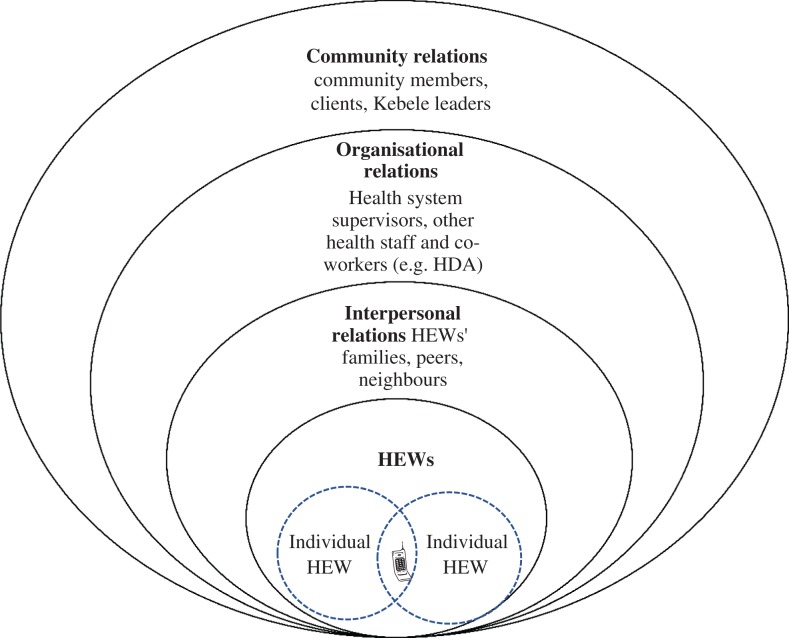
HEWs’ multiple roles and relationships, adapted from McLeroy *et al.* 1988

In line with the WHO framework, our results explore HEWs’ experience of the inequalities generated by unequal gender norms, roles and relations, in order to understand how to address and change underlying power relations. Our results position HEWs at the interface of health systems and communities from where they negotiate a complex range of relationships and power dynamics. A socio-ecological model adapted from McLeroy *et al.* 1988, illustrated in Fig. 3, informs our analysis and frames our results.^27^ The introduction of mHealth shapes these relationships in new and complex ways. Relationships and behaviour, although illustrated in Fig. 3 as distinct from one another, can span more than one sphere. The results are presented starting from the relationships among the HEWs, and then on to different forms of interpersonal relationships, organizational relationships, and finally community level relationships.

### Impact at individual level

Participants spoke of the limited access women in their communities had to mobile phones, which they attributed to their lack of decision-making power. Although all HEWs come from the communities they serve, when they speak about women in their communities, they often set themselves apart in terms of their decision-making ability. This assertion was influenced by HEWs’ education, paid employment and the phone.

A woman living in our community is uneducated and she cannot decide everything like I can. Our culture even does not allow her to decide. Moreover, there is still a negative attitude, that is woman cannot participate in meetings or discussion. [HEW, IDI]

The intervention helps HEWs be more accurate in their data collection and reporting and reminds them to follow-up with patients and clients. HEWs see it as a helping hand and a means to upgrade their skills and knowledge. Many reported increased confidence, more participation in *woreda and kebele* meetings, greater oversight of the data and improved quality.

[O]ur participation rate increased… [due to] the presence of this mobile service…[T]here is great variation in the participation rate from HEWs who work in *Kebeles* where there is no mobile service program. [HEW, IDI]

My commitment to my work is improved. The data quality is improved, I develop self-confidence in my work, and the skill of using the technology is improved. My data handling is also improved. I can say the phone is my friend. [HEW, IDI]

Several HEWs perceived the mobile as an additional person, friend or boss who helped them in their work. This was especially pronounced for HEWs working alone at health posts.

HEWs unable to partake in training, due to maternity/educational leave, felt that their trained counterparts had more opportunities to input into meetings. This suggests that training on the smartphone, rather than the presence of, or primary access to, the smartphone is the defining factor for increased participation in meetings. Drawbacks of the intervention were also identified. Some HEWs reported that they spent extra time inputting data, as paper-based reporting was still required. This was partly because the intervention had not been scaled-up and partly because it focused only on TB and maternal health, excluding the other 14 essential packages that HEWs deliver. The intervention added to HEWs’ work burden and some HEWs, and their supervisors, reported that it also changed the workload divisions and thus the relationships between HEWs.

It is not helpful. Because the one who is trained to handle the mobile is not performing other jobs, once she starts to fill information in mobile…It is not only the mobile text that support the mothers…, rather [it is] our knowledge and skill. [HEW, FGD]

The one who doesn’t have the mobile phone may consider herself inferior to her colleague. [Supervisor, FGD]

Financially, the phones may also inadvertently burden HEWs as they continue paying for the airtime charges. Primarily the HEWs seemed happy to do this as they considered the phones to be their personal property, using the airtime for personal calls and data. Though HEWs also reported being fearful that, in the case of a lost or stolen phone, they would have to bear this cost; which could put additional pressure on their limited financial resources.

I take great care of this mobile…Additionally, if it is lost, it is said that there should be paid by the one who lost it and this is practiced in somewhere. For this reason I feel discomfort. However, I felt great satisfaction getting it, currently I am using it to call. [HEW, IDI]

### Impact on interpersonal relationships

Most HEWs reported an intrinsic desire to help their communities as their reason for joining the HEP, though many also desired ‘an occupation’, ‘a monthly salary’ and noted that HEW ‘occupation is good especially for females’. Many respondents lacked information about the HEW role and workload when joining, with some suggesting they would not have joined had they known the work burden. In Ethiopia, HEW work is in addition to a domestic work burden.

[A woman] starts her routine early in the morning and continues working till night and no one understands her problem. …She prepares food, feeds their children, caring for her children and cleans the compound. She cannot feed herself properly even. [HEW, IDI]

HEWs find this dual burden onerous but stress that they are motivated by the positive results in their communities, the introduction of mhealth and their paid employment.

HEWs serving in rural areas report that male household heads commonly own phones, as men have their own incomes. HEWs are uniquely positioned: as women and wives, with their mainly subordinate status, and as paid workers, with a slightly higher status. The smartphones elevated the HEWs’ status because – although most HEWs owned a phone prior to the intervention – it was not usually a smartphone.

HEWs [were] delivered with better quality of mobile compared to their own [personal phones]… for this reason HEWs working in non-project areas…are saying to HEW working at project area ‘you own a special quality mobile, why it is delivered only for you?’…Our HEWs are glad to have this mobile, because they feel as if this increased their social status…[Project Supervisor, IDI]

One supervisor reported a husband’s appropriation of the phone, however overall HEWs controlled these phones. Thus, when asked about smartphone access, the power dynamic of men as mobile phone gatekeepers was inverted – at home HEWs managed these phones and they reported not allowing their husbands or children access. They were proud of the phones, seeing them as their own, but also as an extension of government property. This indicates some gender transformative attitudes and enhanced HEW status.

I never permit him [my husband] even to touch it. Due to this reason, sometimes he says ‘what kind of mobile [is it that] you have [been] given?!’ [HEW, IDI]

### Impact on relationships with supervisors

The introduction of mobile phones may have resulted in HEWs’ increased collaboration with supervisors. There were reports of a pragmatic, ‘teamwork’ approach: when phones were lost, or network connectivity was down, supervisors would collect and upload the data.

It was lost… I informed to supervisor and currently he is feeding data. [HEW, IDI]

HEWs without primary access to the smartphones also relied on supervisors to address technology skill gaps. Supervisors, in turn, noted the improved quality of HEWs’ data and seemed impressed by HEWs’ ability to adapt to the mHealth system.

I observe that they have positive attitude and the motivation of the HEWs towards their work is improved. They have good attitude towards us too. We are helping them, and we have friendly relationship. [Project Supervisor, IDI]

Supervisors also took more responsibility for monitoring pregnancies and motherhood in the community:

When the message alarm [for a] particular woman comes to them after entering data in mobile, they feel great satisfaction. This is common not only for HEWs but also us. Sometimes the message comes to myself and it feels good so I go to community to follow up those pregnant women. [Project Supervisor, FGD]

Government supervisors, without smartphone access, felt their lack of skills made it harder to appropriately support HEWs who needed help. This, combined with supervisors’ desire to assert their superior skill, knowledge and status, may have a negative impact on their relationships.

HEWs have good skills using this technology, but supervisors do not have skill. Supervisors should have at least one step better skills on this technology compared to HEWs. It is better to give the mobile phone to supervisors and the rest of HEWs to exchange information…. [Government Supervisor, FGD]

### Impact on organizational relationships

Additional intra-cadre power differentials arose after the introduction of one smartphone per health post. HEWs perceived the smartphone as gifted to the health post for good performance and it was generally ‘adopted’ by the more senior HEW, as a signal of status. The senior HEW treated the smartphone as her own property, taking it home each evening. One HEW was said to have retained the phone during maternity leave, demonstrating strong personal ownership. This meant that HEWs ‘without’ smartphones were unable to upgrade their skills. Despite initial training, they had fewer opportunities to use the smartphones.

Yes, for example she [the senior HEW] increased her knowledge and skill about the mobile and also she increased her own work performance. Additionally, she can use Facebook as well as she can take a picture. [HEW, IDI]

Tensions over smartphone usage, ‘ownership’ with connotations of status thus emerged between HEWs. All HEWs and, in some instances, HEWs’ family members, this tension.

She felt uncomfortable with not getting this mobile. Not only for her, but also her husband. I myself too [would feel this way] if [it were me] in place of her. [HEW, IDI]

### Impact on relationships with community members

HEWs believed that the Smartphones increased communities’ recognition of their status and that this was not dependent on phone ownership.

… They are happy while we follow-up with mothers after feeding their data in this mobile. And mothers give great respect to us. [HEW, IDI]

Q: Does the community give special respect for your colleague after she got phone?

A: The communities are giving respect for both of us in the same way. Only I and she have information about the mobile phone including its purpose. [HEW, IDI]

Although HEWs stressed the shared status, their own accounts of smartphone usage demonstrate that status is linked to the smartphones. A *kebele* leader similarly suggested that the HEW in possession of the mobile is perceived by the community to have a higher status.

Additionally, one HEW reported that smartphones led to greater community expectation which could inadvertently place additional stress on the HEWs.

Q: What is the feeling of the community on the mobile phone?

A: They are happy, encourage us to work hard more and they are giving positive comments since we get this chance, so they expect more things. [HEW, IDI]

## Discussion

### Main finding of this study

HEWs’ lives are embedded within the communities they serve. As individuals they have a unique role and agency to shape health outcomes. Like other women health workers, they juggle multiple workloads; undertaking employed health work alongside household and childcare responsibilities.^17,28,29^ Adapting the McLeroy *et al.* 1998 socio-ecological model, this study explored the experience of HEWs interface role with regard to mHealth’s impact on their work and on relationships within households, communities and in the health system. In parallel, applying the WHO framework demonstrates that while the mHealth intervention did change gender and power relations, it did not address the underlying causes of gendered health inequalities. In this sense, it did not achieve the WHO milestone for gender transformative interventions. Rather, the intervention intentionally addressed gender-based health inequalities through remedial action. This, in accordance with the WHO framework, is a ‘gender specific’ approach and intervention – a step towards gender transformative research, but not yet there. Incremental improvements nonetheless result in better healthcare provision for populations. As demonstrated here, the intervention equipped HEWs with knowledge and tools to perform effectively and provide more equitable care to communities; reflecting mHealth’s positive potential for health outcomes and health system strengthening.^2,5,12^

These findings demonstrate the increased social status and agency felt by the HEWs, who as government employees, already experience superior status; they acted as gatekeepers to the phones within households inverting the traditional patriarchal norms where men are the primary keepers of technology. In most cases, HEWs felt the smartphones improved their skills; gave them opportunities to share their knowledge in meetings; and aided their workloads by acting as a ‘boss’ or a ‘friend’, serving helpful reminders to follow-up with patients. However, the unequal distribution of the smartphones also changed power dynamics between HEWs, impacting on workload distribution. Inadvertent financial burdens linked to the running costs and potential loss or theft of the mobiles introduced new pressures on the HEWs. These drawbacks require consideration for scale-up.

### What is already known on this topic

Literature on mHealth interventions and patient empowerment shows mHealth has the potential to empower communities and transform harmful gender norms but access and use may also reflect and extend current gender inequities.^12^ A 2013 review looking at studies from Nigeria, India, Tanzania, Uganda and the Congo found that in cases where husbands did not have access to phones, female community members would render phones to husbands.^12^ However, our findings show a shift in patriarchal norms – HEWs became the gatekeepers of the technology, not allowing their husbands access. Similarly, an Indian intervention^30^ which registered CHWs phones in women’s names, led to male household members requiring permission to handle the phones.

Our findings echo concerns in the literature about mHealth financing of HEWs’ smartphones. By not providing unlimited airtime, health extension programmes risk transferring the financial burden to those least able to afford it. Hampshire *et al.* argue that CHWs subsidize health care from their own pockets when expected to pay mobile airtime.^5^ This links to wider debates in moral economies of care, which sees women undertake a large proportion of the unpaid care workforce. Maes (2015) describes the institutional rhetoric of urban Ethiopian CHWs as ‘priceless’.^31^ This rhetoric is internalized by CHWs, who feel a strong moral obligation to care for the sick or pregnant in their communities at their own expense,^5,31^ at times further impoverishing themselves and their families.^32^

Conversely, financing smartphone usage could further limit informal use. Although no restrictions were issued, some HEWs refrained from personal gain as the smartphones were government property. Given Ethiopia’s political climate during data collection, which had seen social media use restricted in the wake of anti-government protests,^33^ it’s possible that HEWs felt uncomfortable using government devices beyond their official capacity. While there is no evidence as to whether women are more likely to be affected by the political climate, our results demonstrate proud women over-burdened in their work and limited in their choices. HEWs are low in health system hierarchy^34^ and they saw their jobs as ‘good for females’, but arguably the litmus test for gender transformative programmes is that HEW employment becomes acceptable as men’s work with attractive employment conditions.^17,35^

Medhanyie and colleagues found, in Tigray, Northern Ethiopia, that HEWs’ unrestricted smartphone usage helped familiarize and motivate HEWs.^36^ Unrestricted use may have multiple benefits to healthcare – in Kenya CHWs’ Whatsapp groups disseminated health information at times of outbreak, built morale, improved supervision and documented the quality of services delivered.^37^ Moreover, HEWs in Tigray used smartphones for accessing the internet and social media, thus independently gaining information and resources.^36^

### What this study adds

Many studies have focused on communities’ gendered access to mobile technology^12^ whereas this paper examines mHealth’s impact on the experiences of female HEWs. Unlike in Bangladesh, where technology was appropriated by husbands or seniors as a consequence of gender dynamics,^2,12^ our findings show signs of change in household dynamics in a context where, although phone ownership was a male norm, HEWs have become the gatekeepers of the phones accorded to them.

Application of the WHO framework for gender transformative research demonstrates positive HEW empowerment in skill building and data handling. The intervention increased HEWs’ expectations of themselves and communities’ expectations of HEWs. This is not unique to Ethiopia. In Malawi and Ghana, CHWs’ mobile phones led to additional time and emotional burdens, with CHWs often responding to out-of-hours calls.^5^ These burdens, along with the risk of theft and loss – also reported as a concern of Mozambique’s CHWs^7^ – show smartphones add status and help HEWs perform well on the one hand and increase risks on the other. HEWs, as women, may have diminished ability to endure such risks as they have fewer resources and networks.

HEWs face large workloads and technological interventions should support, rather than undermine. In this study, changed power dynamics and some tensions were reported between HEWs as one of the HEWs adopted the phone for her personal use. While tensions over phone ownership and phone-related activities affecting work burdens have been reported between couples,^12^ to our knowledge this has not been reported within health system cadres. This is an example of how, even with the best of intentions, there is still opportunity for gender and power dynamics to play out in unexpected ways.

### Limitations of this study

First, our study focused on a relatively small subset of HEWs in one region of Ethiopia and this context may differ from other parts of the country.^38^ Secondly, as this intervention was a pilot, we could not explore how mHealth technology played out in gendered ways across all health packages. Thirdly, we must consider our positionality as researchers. While every effort was made to ensure participants understood the confidentiality and were able to speak openly, it may be that they saw the (local and otherwise) data collectors as project staff, government workers or ‘outsiders’ and tailored their answers accordingly.^39^ It is also important to situate our findings within the political context of Ethiopia, which may limit freedom of speech during the study period.^40^

## Conclusion and recommendations

Although intended to enhance female HEWs’ role in Ethiopia’s health system, introducing technology without addressing power relations or other dimensions of their work can bring challenges. Specific actions could make the intervention more gender transformative: distribute smartphones to all HEWs to avoid creating inequalities; ensure workloads are equally shared and that all HEWs are given opportunity to upgrade their skills. Additionally, scale-up of the intervention may alleviate HEWs’ workload and build their skills as data collection is streamlined across all 16 health packages. However, this will require further research and technical support for troubleshooting that, while manageable in a pilot, may cause delays at scale.

Supportive policy change that fosters progressive changes in the underlying power relations and in the structure of the health system should challenge patriarchy in the household, community and health system. It should recognize women’s rights as individuals; challenge norms that equate household and reproductive work to women’s work; create opportunities for HEWs to engage in policy-making processes;^34^ and enable progression to more senior positions, such as that of supervisor. Failure to address these dimensions may mean that HEWs’ mobile phones reproduce a cultural gender imbalance that may be holding this cadre back.

## Appendix

### Appendix 1: Interview Topic Guides in English

**Topic Guide – In-depth interview guides for HEWs in Ethiopia**

1. Introduction:

***Thank you for taking the time to speak to me. I would like to understand a little bit more about the mobile phone programme that has recently been implemented for HEWs in your district and how this has impacted your work and life outside of work. If you don’t want to answer any of the questions in the interview please let me know and we can move on to something different. If any of the questions are unclear please tell me and I am happy to ask them in a different way.***

**Introductory questions/background**:

- Age:
- Education/years of school:
- Can you describe your household?
  o Who do you live with? (Husband? Children? Parents?)
  o What are your duties around the house?/what responsibilities do you have at home?
    ▪ Does anyone help with these duties?
- Can you tell me a bit about your community?
  o Have you always lived in this community?
  o If no, where did you live before?
    ▪ Why did you move?
    ▪ How was the community different to this one?
- Can you tell me about how you became a HEW?
  o How long have you been a HEW?
  o Why did you become a HEW?
  o Have you ever wanted to leave or do anything else?
    ▪ If yes, what is currently stopping you from engaging with these activities?

**Logistics of the phone use**

- How have you found the mobile phones you have been issued?
  o Have you found it easy to use?
    ▪ If so in which ways?
    ▪ Do you think it’s successful so far?
  o Have there been any technical issues or problems with using it?
    ▪ If so, how?
    ▪ Has this had any impact – i.e. damaged relationship with the health system or the community?
- Can you tell me how the airtime is managed?
  o Have there been any issues with this process?
  o If so, do you think there is any impact of this?
- How do you charge the phone?
  o Is it convenient to do?
  o Have you ever been without charge when visiting a client, or has it ever impacted your work? e.g. delayed reporting to health system

**Access to phones**

- Do you or anyone else in your household have a personal phone?
  o If so who – yours/husband/mother etc?
  o Did you have access to a mobile phone prior to this?
    ▪ If so, was it yours?
      – If not, whose was it specifically?
    ▪ Do you use it differently to the phone used for work purposes?
    ▪ Does anyone else use your phone?
      – If so, who?
  o Generally, do households in your community have access to phones?
    ▪ If so who holds the phone?
    ▪ Do your (female) clients have access to phones?
    ▪ If so, are they personal or household phones i.e. owned by someone else in the family or community?

**Impact on work**

- How do you feel having this resource available to you?
- Has the phone impacted your work at all?
  o Do you use it for SMS/calls/reporting
  o Has the technology enabled you to be more/less accurate in your work?
    ▪ If so please expand
  o Has your reporting to the health system changed from using the phone?
    ▪ Reporting more/less often than before?
    ▪ Any other methods of reporting (for TB and MNCH)?
- Has it impacted your relationship with your supervisor?
  o If so can you expand? In what ways has it changed and how do you think the mobile is responsible for this change?
  o Is your supervisor male or female?
    ▪ If male, have you always had a male supervisor?
    ▪ Do you like having a male?
    ▪ What do you think it would be like having a female supervisor? (Would it change anything for you?)
- Has the phone has impacted on your relationship with your community?
  o Probe how/why
- Has the phone has impacted on your relationship with your colleagues, for example other health extension workers, supervisors, or any one else at woreda level?
  o Probe how/why
  o When there are meetings (at woreda level) do you actively participate?
    ▪ If no, why not, would you consider it?
    ▪ Who mainly does participate
    ▪ If yes, did you always participate?
    ▪ Has the phone changed your level of engagement/participation?
    ▪ Have you noticed a change in the general engagement of HEWs since the phone program came into use?

**Unintended consequences**

- How do you feel having this as a resource in your personal life?
- How do you use the phone outside of your work duties?
  o Probe – apps/social media/text messages/camera/calls to friends etc.
    ▪ If so probe exactly in which ways and how often?
    ▪ Do you meet with friends, other women in your community/other communities?
    ▪ If so please can you elaborate?

- How has this impacted your personal life?
  o For example as women, do you feel it has changed your place in the community (social status)?
  o Have you experienced any negative consequences from having the phone?
    ▪ If yes, can you tell me more about that?
      – E.g. Have you ever felt someone may be jealous because you now have this phone for your work?
      – If so, probe further, have you ever felt threatened or in danger due to the phone?
    ▪ If not, why not?

Thank you so much for time. Is there anything else you want to share with me?

**Topic guide – In-depth interview guides for Supervisors in Ethiopia**

Introduction – Supervisors:

***Thank you for taking the time to speak to me. I would like to understand a little bit more about the mobile phone programme that has recently been implemented for HEWs in your district and how this has impacted your work and life outside of work. If you don’t want to answer any of the questions in the interview please let me know and we can move on to something different. If any of the questions are unclear please tell me and I am happy to ask them in a different way.***

**Reasons for becoming a supervisor**

- Are you from this community?
  o Can you tell me a little about the community?
- How long have you been a supervisor?
- Did you take part in mobile phone training?
  o Is ongoing training offered if needed?
- What are the main things you like/dislike about your role?

**Logistics of the phone use**

- How have the HEWs adapted to using the mobile technology in your opinion?
  o Probe for positives/negatives
  o Have there been any technical issues or problems with using it?
    ▪ If so, how?
    ▪ Has this had any impact on relationships with the health system or the community?
  o Do you think it is successful so far?
- Do you know how the airtime is managed?
  o Does this run smoothly?
    ▪ Have there been any issues with this process?
    ▪ If so, do you think there is any impact of this?

**Impact on work**

- How do you think the phone has impacted on the work of the HEWs?
- Can you tell me about their attitudes to the phone and to work?
  o Have you noticed this has changed at all?
  o In what way?
- Have you found the reporting to have changed at all?
  o In what way – accuracy? Timeliness?
    ▪ Why do you think this is the case?
    ▪ Is this the only way reporting occurs (for TB and MNCH)?
  o Has it had any impact on your relationship with your supervisees?
    ▪ Do you communicate using the phone? Arranging meetings e.g. catchment area meetings
    ▪ If so can you expand? In what ways has it changed and how do you think the mobile is responsible for this change?
  o How do you think the phone has impacted on your supervisees relationships with other health extension workers, or any one else at woreda level?
    ▪ If so can you expand?
    ▪ When there are meetings held (at woreda level) do you actively participate?
      – Probe here.
      – Who mainly does participate?
    ▪ Do your supervisees actively participate?
      – If no, why not?
      – If yes, did they always participate?
      – Has the phone changed their level of engagement/participation?
      – Have you noticed a change in the general engagement of HEWs at meetings since the phone program came into use?
- Do you think your gender has an impact on your relationship with the HEWs you supervise?
  o If male: How do you think it might be different with a female supervisor?
    ▪ Probe – why
  o If female: How do you think it might be different with a male supervisor?
    ▪ Probe – why

**Unintended consequences**

- Do HEWs use the phones outside of work?
  o What for? Probe – apps/social media/text messages/camera/calls to friends etc.
    ▪ Probe exactly in which ways and how often?
  o If so, do you think there are any positive consequences of this?
    ▪ E.g. do you think it may have changed HEWs place in the community (social status)?
    ▪ Or their place within the health system, e.g. do they have more of a voice in meetings (at woreda level)? With other colleagues etc?
  o Have you ever heard of any negative consequences of the mobile phone use?
    ▪ At woreda level?
    ▪ At community level?
    ▪ At household level?
    ▪ If so, probe further – exactly what
      – Jealously, crime etc.
    ▪ If not, why not?

Thank you so much for time. Is there anything else you want to share with me?

**Topic Guide – Focus Group Discussions with Health Extension Workers**

1. Introduction:

***Thank you for taking the time to speak to me. I would like to understand a little bit more about the mobile phone programme that has recently been implemented for HEWs in your district and how this has impacted your work and life outside of work. If any of the questions are unclear please tell me and I am happy to ask them in a different way. You are free to leave at any time.***

**Introductory questions/background**:

- How long have you been a HEW?
- Why did you become a HEW?
- Have you ever wanted to leave or do anything else?
  o If yes, what is currently stopping you from engaging with these activities?

**Logistics of the phone use**

- How have you found the mobile phones you have been issued?
  o Have you found it easy to use?
    ▪ If so in which ways?
    ▪ Do you think it’s successful so far?
  o Have there been any problems with using it?
    ▪ If so, how?
    ▪ Has this had any impact on your relationship with the health system or the community?
- How do you manage the airtime?
  o Have there been any issues with this process?
  o If so, do you think there is any impact of this?
- How do you charge the phone?
  o Is it convenient to do?
  o Have you ever been without charge when visiting a client, or has it ever impacted your work? (e.g. delayed reporting to health system)

**Access to phones**

- Do households in your communities have access to phones?
  o If so who holds the phone?
  o Do your (female) clients have access to phones?
  o If so, are they personal or household phones i.e. owned by someone else in the family or community?

**Impact on work**

- How do you feel having this resource available to you?
- Has the phone impacted your work?
  o How do you use it outside of inputting client data? E.g. calls/SMS/arranging meetings?
  o Has the technology enabled you to be more/less accurate in your work?
    ▪ If so please expand
    ▪ If not, why?

- How often do you report back to the health system using the phone?
  o Is this more/less often than before?
  o Is this the only way you report now (for TB and MNCH)?
- Has it had any impact on your relationship with you supervisors?
  o If so can you expand? In what ways has it changed and how do you think the mobile is responsible for this change?
  o Do any of you have female supervisors?
    ▪ If yes, do you think this is different from those with male supervisors?
      – If so, how?
    ▪ If male, have you always had a male supervisor?
    ▪ Do you think having a female supervisor would change anything for you in your work?
- Has the phone had any impact on your relationship with your communities?
  o If so can you expand?
  o Has the phone had any impact on your relationship with your colleagues at woreda level?
    ▪ If so can you expand?
    ▪ When there are meetings (at woreda level) do you actively participate?
      – If **no**, why not, would you consider it?
      – Who mainly does participate
      – If **yes**, did you always participate?
      – Has the phone changed your level of engagement/participation?
      – How about other HEWs at the meeting?

**Unintended consequences**

- Do you use the phone outside of your work duties?
  o How do you feel having this as a resource in your personal life?

- What do you use it for?
  o Probe – apps/social media/text messages/camera/calls to friends etc.
    ▪ If so probe exactly in which ways and how often?
    ▪ Do you meet with friends, other women in your community/other communities?
    ▪ If so please can you elaborate?
- How do you feel this has impacted on your personal lives?
  o In either a positive or negative way?
  o For example as women, do you feel it has changed your place in the community (social status)?
  o Have you experienced anything negative from having this resource?
    ▪ If yes, can you explain futher? E.g Have you ever felt jealousy because of this new resource?
    ▪ If so, probe further, have you ever felt threatened or in danger due to the phone?
    ▪ If not, why not?

Thank you so much for time. Is there anything else you want to share with me?

**Topic Guide Focus Group Discussions with Supervisors**

2. Introduction – Supervisors:

***Thank you for taking the time to speak to me. I would like to understand a little bit more about the mobile phone programme that has recently been implemented for HEWs in your district and how this has impacted your work and life outside of work. If you don’t want to answer any of the questions in the interview please let me know and we can move on to something different. If any of the questions are unclear please tell me and I am happy to ask them in a different way.***

**Logistics of the phone use**

- How do you think the HEWs have adapted to using the mobile technology?
  o Do you think it is successful so far?
  o Have there been any problems with using it?
    ▪ If so, how?
    ▪ Has this had any impact on relationships with the health system or the community?
- Do you know how the airtime is managed?
  o Have there been any issues with this process?
  o If so, do you think there is any impact of this?

**Impact on work**

- What effect has the phone had on the work of the HEWs?
  o Has it impacted your work life?
- What is their attitude to the phone/work?
  o Do you think this resource has changed their attitudes to work at all?
- Have you found the reporting to have changed at all?
  o In what way – accuracy? Timeliness?
    ▪ Why do you think this is the case?
    ▪ Is this the only way reporting occurs (for TB and MNCH)?
- Has it had any impact on your relationship with your supervisees?
  o If so can you expand?
  o In what ways has it changed and how do you think the mobile is responsible for this change?
- From your impression, do you think the phone had any impact on your supervisees relationships with other health extension workers, or any one else at woreda level?
  o If so can you expand?
  o When there are meetings held (at woreda level) do you actively participate?
    ▪ Probe here.
    ▪ Who mainly does participate?
  o Do your supervisees actively participate?
    ▪ If no, why not?
    ▪ If yes, did they always participate?
    ▪ Has the phone changed their level of engagement/participation?
    ▪ Have you noticed a change in the general engagement of HEWs at meetings since the phone program came into use?
- If male group: do you think your gender has an impact on your relationship with the HEWs you supervise?
  o Probe, how?
  o Why do you think that is?
- Do you think it might be different with a female supervisor?
  o How, why?
- If mixed group: do you think HEWs have a preference for a male/female supervisor?
  o Why do you think that is?

**Unintended consequences**

- Are you aware of the HEWs using the phones for personal use (outside of work)?
  o If so for what?
  o Probe – apps/social media/text messages/camera/calls to friends etc.
    ▪ If so probe exactly in which ways and how often?
  o If so, do you think there are any positive consequences of this?
    ▪ E.g. Do you think it may have changed HEWs place in the community (social status)?
    ▪ Or their place within the health system?
      – E.g. do they have more/less of a voice in meetings (at woreda level)? With other colleagues etc?
  o Have you ever heard of any negative consequences of the mobile phone use?
    ▪ If so, probe further
    ▪ If not, why not?

Thank you so much for time. Is there anything else you want to share with me?

**Topic Guide Focus Group Discussions with Community Leaders**

Introduction – Community leaders

***Thank you for taking the time to speak to me. I would like to understand a little bit more about the mobile phone programme that has recently been implemented for HEWs in your district and how this has their role and status in society. If you don’t want to answer any of the questions in the interview please let me know and we can move on to something different. If any of the questions are unclear please tell me and I am happy to ask them in a different way.***

**Perception of HEWs**

Firstly can you tell me a bit about how women become HEWs in your *kebele*?

– Probe: selected from community, self-selecting?

Do you think it is seen as a desirable job?

– Probes- why?

Can you describe the perception of HEWs in this community?

– Probes: Do you think that everyone views them in this way, or could men/ /women/children perceive them differently? If so, in what ways – please describe?
– How does this compare to other women in the community?
– How does this compare to men in community?
– Are HEWs valued in community?
  o Probe – Why?

Are HEWs supported in their work?

– by community members
– by health system
– Or in other ways?

**Impact on work**

Are you aware of the mobile phones that HEWs in your *kabele* are now using in their work? (If not briefly explain project and ask can ask the questions hypothetically)

What impact do you think this is having on their work, if any?

– Probe on community perception of the work they are doing and how this may have changed since using the phones

Are you aware of any changes in workload for the HEWs?

– If yes- when did it change? what is the reason?
– Please can you describe how it has changed?

Are you aware of the communities’ impressions of the phones for HEWs?

– Probe: is their role seen as more/less important/unchanged
  Have HEWs links to the health system changed in any way?
  o If so, probe – How? Due to what?

Thinking about their place within the community (and health system), to what extent to HEWs participate in *Kabele* meetings?

– How does their involvement compare to other women in the community?
– Probe: has this changed since the phones came into use?

What is your perception of career advancement opportunities for HEWs?

– Do you think the phones have any impact on this?
– If not, why not?
– If so in what ways? Stronger links to health system, motivation etc?

**Unintended consequences**

- Are you aware of the HEWs using the phones in their personal life/outside of work?
  o If so for what?
  o Probe – apps/social media/text messages/camera/calls to friends etc.
    ▪ If so probe exactly in which ways and how often?
  o Do you think there are any positive or negative consequences of having the phones outside of their distinct roles?
    ▪ If so, probe further
    ▪ If not, why not?

Thank you so much for time. Is there anything else you want to share with me?
